# Respiratory Health in Adults Residing Near a Coal-Burning Power Plant with Coal Ash Storage Facilities: A Cross-Sectional Epidemiological Study

**DOI:** 10.3390/ijerph16193642

**Published:** 2019-09-28

**Authors:** Abby N. Hagemeyer, Clara G. Sears, Kristina M. Zierold

**Affiliations:** 1 Department of Epidemiology and Population Health, University of Louisville, Louisville, KY 40292, USA; 2 Department of Epidemiology, Brown University, Providence, RI 02912, USA; 3 Department of Environmental Health Sciences, School of Public Health, University of Alabama at Birmingham, Birmingham, AL 35294, USA

**Keywords:** coal ash, fly ash, coal combustion residuals, coal-burning power plants, respiratory health, air pollution, environmental health

## Abstract

Coal ash, the byproduct of burning coal made up of small particles, including heavy metals and radioactive elements, is discarded in open-air landfills where it can be emitted into the air, contributing to air pollution in the surrounding community. Few regulations exist regarding the storage, disposal, and transport of coal ash. There is limited research on the health impacts of coal ash exposure on communities. The purpose of this study was to examine the prevalence of respiratory symptoms among adults exposed to coal ash and non-exposed adults. A cross-sectional epidemiological study was conducted among two populations: one exposed to coal ash and one not exposed to coal ash. Perception of health (*p*-Value < 0.0001), cough (Adjusted Odds Ratio (AOR) = 5.30, 95% Confidence Intervals (CI) = 2.60–11), shortness of breath (AOR = 2.59, 95% CI = 1.56–4.31), hoarseness (AOR = 4.02, 95% CI = 2.45–6.60), respiratory infections (AOR = 1.82, 95% CI = 1.14–2.89), and mean overall respiratory health score (*p*-Value < 0.0001) were all statistically significantly greater in exposed adults (*N* = 231) when compared to non-exposed adults (*N* = 170). Adults residing near the coal ash facility were more likely to report respiratory symptoms than the non-exposed population. More research on the health impact of coal ash and storage regulations needs to be conducted.

## 1. Introduction

Coal is a combustible sedimentary rock that is largely formed from plant debris [1]. It is made up of carbon, hydrogen, oxygen, nitrogen, and sulfur, as well as small amounts of various metals and radioactive materials [1,2]. Coal is mined to meet the needs of the world’s increasing demand for fuel. Despite efforts to increase natural gas production, coal combustion power plants continue to play a major role in electricity production [1,3]. Coal-burning power plants generate approximately 27% of the electricity in the United States [4].

Coal combustion residuals (CCR), the byproduct of coal combustion, consist of small particles that contain naturally occurring radioactive materials, polycyclic aromatic hydrocarbons, and a variety of heavy metals, including aluminum, arsenic, iron, lead, and mercury [5,6,7,8,9,10,11,12]. In 2017, the United States produced nearly 111 million tons of CCR, more commonly known as coal ash [13]. Coal ash is not coal dust, which is the term used to refer to a type of pollution generated from the mining of coal. The composition and particle size of coal ash are different compared to coal dust.

Coal ash is an overarching term that can include flue gas desulfurization solids, boiler slag, bottom ash, and fly ash [3]. During the coal combustion process, small spherical particles measuring ≤10 µm in diameter ascend up the stack and are collected on filters. These small particles are known as fly ash and account for nearly 40%–70% of coal ash product [3,14]. Although some effort is being made to reuse coal ash, in 2017, only 64% of coal ash was recycled and used in products such as cement [13]. The coal ash that is not reused gets stored in designated landfills and ash ponds where it becomes a likely source of pollution [15]. Fugitive fly ash emissions from coal ash storage facilities may be a significant contributor to the concentration of ambient air particulate matter (PM) [16,17].

Although studies on coal ash exposure and human health are lacking, several researchers have investigated the occupational health hazards of coal ash. Two researchers found that coal ash workers exposed to high arsenic levels had higher malignancy-caused death and higher rates of cancer mortality when compared to coal ash workers exposed to “normal” levels of arsenic [18,19]. More recent studies sought to examine occupational health hazards of working at a fly ash treatment plant compared to a bottom ash recovery plant. One researcher found that when compared to bottom ash workers, workers in the fly ash treatment plant had significantly higher plasma malondialdehyde [20]. Another researcher found that fly ash treatment plant workers had more DNA damage when compared to their bottom ash counterparts [21]. Although a few occupational health studies have been conducted, epidemiologic studies investigating the impact coal ash has on the surrounding community are limited [22,23]. Since coal ash landfills and ponds are maintained in residential communities, it is important to understand community exposure.

The purpose of this study was to assess the prevalence of respiratory symptoms and illness among adults living in neighborhoods surrounding a coal-burning power plant with coal ash storage facilities compared to adults living in a non-exposed environment. The exposed population lives in Louisville, Kentucky, an area that is consistently ranked by the American Lung Association in the top 25 cities in the United States for the most particulate matter pollution in the country. We hypothesized that the adults residing in neighborhoods near the coal-burning power plant with coal ash storage facilities would have poorer respiratory health and more respiratory symptoms than adults who are not exposed to coal ash. By characterizing respiratory symptoms in the populations, our analysis begins to fill the gap in the research literature regarding the potential health effects of coal ash among adults.

## 2. Methods

In this analysis, we used a cross-sectional questionnaire which was developed by the research team in conjunction with community members living near the coal-burning power plant. This questionnaire was used to identify respiratory symptoms and health in an exposed and non-exposed comparison population. This study was a community-based mixed methods study (focus groups and a cross-sectional epidemiological study) that took place in four neighborhoods surrounding a coal-burning power plant with coal ash storage facilities in Southwest Louisville, Kentucky [22,23]. The exposed neighborhoods that were included in this study were all within 1/4 to 1/2 miles of the storage facility. The storage facility, located just east of the Ohio River, is home to one large coal ash landfill and five ponds, two of which are known to store coal ash. The landfill was last estimated to have an elevation of over 500 feet and a surface area of 110 acres [24]. The main coal ash storage pond, which is located just 1200 feet from the Ohio River, has a surface area of approximately 40 acres [25]. To compare the prevalence of respiratory symptoms and illness in the coal ash exposed population, a non-exposed population without coal-burning power plants or coal ash storage facilities located in Orange County, Indiana, was chosen.

All methods were approved by the University of Louisville Institutional Review Board (IRB#: 09.0141). Participants were provided a preamble prior to completing the questionnaires. Adults who finished the questionnaire and submitted it to the research team were considered to have consented.

### 2.1. Study Populations

#### 2.1.1. Exposed Group

Exposure was defined as residing in one of the four neighborhoods adjacent to the coal ash facility. The study encompassed a community-based research design in which residents were recruited from four neighborhoods surrounding a large coal-burning power plant. The four neighborhoods included in the study span two zip codes. According to the U.S. Census in 2010, the first zip code region was home to 40,746 people; 63% were white, 76.3% were 18 years or older, 14.3% were 65 years or older. Additionally, the average household size was 2.41. According to the 2010–2014 American Community Survey 5-Year Estimates, 37.9% of those 25 years and older were at least high school graduates; the poverty level among these same individuals was 15.7%.

The 2010 U.S. Census reported that the second zip code region was made up of 26,465 people; 86.6% were white, 75.1% were 18 years or older, 13.6% were 65 years or older, and the average household size was 2.54. According to the 2010–2014 American Community Survey 5-Year Estimates, 44.1% of persons 25 years and older were at least high school graduates, 7.5% were living in poverty.

#### 2.1.2. Non-Exposed Comparison Group

A non-exposed comparison population was chosen from Orange County, Indiana, approximately 60 miles from Louisville, Kentucky. Orange County was chosen because its population has similar demographics and it is rurally situated, without close proximity to a coal-burning power plant or coal ash storage facilities. In 2010, the U.S. Census reported that of the 19,840 residents of Orange County, Indiana, 98.2% were white, 75.4% were 18 years or older, and 15.8% were 65 years or older. Additionally, the average household size was 2.49. Of the people 25 years and older, 45.1% were at least high school graduates and 12.2% were living in poverty.

### 2.2. Collection of Data

Data were collected from a cross-sectional survey of exposed and non-exposed adults. The questionnaire for the survey was developed by the research team based on results from five focus groups that were held with 26 adult community members that lived near a coal-burning power plant with coal ash storage facilities [22,23]. In addition to the health conditions and symptoms that resulted from the focus group discussions, health conditions and symptoms that were known to be associated with exposure to particulate matter were included in the questionnaire. Although the reliability and validity of the questions were not assessed, the questionnaire underwent pilot testing with a group of community leaders who provided feedback on the length, time, and appropriateness of the questions. After the feedback was incorporated, the questionnaire was used for the survey. In order to recruit community members to participate from each of the four neighborhoods, the research team distributed informational flyers to residents in the neighborhoods. The flyers informed potential participants about the study and invited them to participate by filling out the questionnaire. A central location in each neighborhood was chosen and written questionnaires were available on on multiple days for participants to complete on their own. English-speaking adults 18 years and older were included in this study.

The questionnaire used to sample the non-exposed comparison population was adapted in order to survey the comparison group in Orange County, Indiana. All demographic, smoking, health, and symptom questions remained the same, but the questions about coal ash exposure and behaviors related to coal ash were altered into questions about outdoor health and wellness. Research team members traveled to a comprehensive health clinic in the community on multiple occasions to administer the questionnaire. English-speaking adults, 18 years or older, that were visiting the clinic were invited to complete the questionnaire. This study assessed adults because adulthood exposure to particulate matter can contribute to respiratory illness and exacerbate symptoms of chronic respiratory diseases. For example, air pollution exposure increases adulthood risk for both Chronic Obstructive Pulmonary Disease (COPD) and emphysema. Respiratory symptoms can greatly disrupt the quality of life of adults and put them at risk of other health concerns.

### 2.3. Respiratory Symptoms Questions

The questionnaire that was utilized in this study assessed several respiratory symptoms and health conditions. To determine the symptoms that exposed and non-exposed participants reported, we asked “How frequently do you experience: cough, shortness of breath, hoarseness, and respiratory infections”. Responses consisted of never, sometimes, and frequently. For this analysis the symptoms were dichotomized as “never” and “sometimes/frequently”. To determine whether a participant had asthma, allergies, or other lung diseases, we asked “Have you ever been told by a doctor or health care provider that you have (circle Y if Yes)”. Participants who were told by their health care professional that they had these health conditions circled a “Y”. For this analysis, health conditions were coded as “1” for yes and “0” for no.

### 2.4. Data Analysis

Data were statistically analyzed using SAS 9.4 (SAS Institue Inc., Cary, NC, USA). The data analysis included a total of 401 questionnaires from participants; 231 completed questionnaires from the exposed population and 170 questionnaires from the non-exposed comparison population. SAS 9.4 was used to examine the prevalence of demographic characteristics and general behaviors of the study populations. The median age of participants in the exposed and non-exposed groups was compared using a Wilcoxon median two-sample test. For categorical demographic variables, Pearson’s chi-squared test was used to compare the proportion of the selected variable between the exposed group and non-exposed comparison group. Differences in the prevalence of self-reported respiratory symptoms and health conditions, including cough, shortness of breath, hoarseness, respiratory infections, asthma, allergies, and other lung conditions, were assessed between the exposed and non-exposed groups using Fisher’s exact test or chi-square tests. Logistic regression was utilized to calculate odds ratios. First, non-adjusted odds ratios and 95% confidence intervals from simple models with just the symptoms and exposure were determined. Then, final models that were adjusted for gender, age, and years of smoking, were used to report adjusted odds ratios, and 95% confidence intervals. The Cochran–Armitage two-sided trend test was used to assess if participants who spent more time outside were more likely to experience respiratory infections.

An overall respiratory health score was created based on self-report of all respiratory symptoms: cough, shortness of breath, hoarseness, respiratory infections, and asthma, and other lung diseases. Variables were dichotomized as “0” for never responses and “1” for sometimes or frequently responses. The overall respiratory health score reflects a numeric score for the number of positive responses of respiratory symptoms. The greater the reported respiratory symptoms the higher the overall respiratory health score. A Wilcoxon Rank-Sum test was used to compare the mean score for the exposed versus non-exposed comparison group.

## 3. Results

In the exposed population, 49% of participants lived in their home for >20 years, 30% lived in their homes for 5–20 years, and 20% lived in their home for <5 years. In the non-exposed population, 23% lived in their homes for >20 years, 36% lived in their homes for 5–20 years, and 42% lived in their homes for <5 years. Table 1 describes the demographics and general behaviors compared between the exposed and non-exposed populations. Age, gender, and years smoked were all statistically significantly different (*p*-value < 0.05) between the exposed and non-exposed groups.

The exposed population had a more even ratio of female to male participants, while nearly three-fourths of the non-exposed population was female. Additionally, the exposed population was slightly older, median age was 52 years compared to 43 years for the non-exposed population. While the exposed population reported having smoked longer compared to the non-exposed population, other general behaviors like current smoker status and time spent outside were similar between the two populations.

The population exposed to coal ash was more likely to report having a poorer perception of health. For example, 46.8% of the exposed population perceived their health as poor or fair (16.2% and 30.6%, respectively), compared with only 28.8% of the non-exposed population (10.2% and 18.6%, respectively). The non-exposed population was more likely to perceive their health as very good or excellent 37.7% compared to 11.4% of the exposed population.

In addition to a poorer perception of health, the exposed group also more frequently experienced several respiratory irritations and illnesses (Table 2).

The exposed population was more likely to report frequent coughing compared to the non-exposed comparison group (36.8% vs. 9.2%, *p*-Value < 0.0001). The exposed group was also more likely to report frequent shortness of breath (33.6% vs. 9.1%, *p*-value < 0.0001), hoarseness (15.9% vs. 1.2%, *p*-value < 0.0001), and respiratory infections (16.8% vs. 2.42%, *p*-value < 0.0001). On average, participants in the exposed group reported more respiratory symptoms than the non-exposed comparison group (mean overall respiratory health score: 3.87 vs. 2.82, *p*-value < 0.0001).

Table 3 reports the results from the logistic regression modeling. Adults who lived near the coal-burning power plant with coal ash storage facilities were more likely to suffer from cough (AOR = 5.3, 95% CI = 2.60–11), shortness of breath (AOR = 2.59, 95% CI = 1.56–4.31), hoarseness (AOR = 4.02, 95% CI = 2.45–6.60), respiratory infections (AOR = 1.82, 95% CI = 1.14–2.89), and allergies (AOR = 1.62, 95% CI = 1.02–2.58).

A gradient response of respiratory infections by time spent outside was assessed in both the exposed and non-exposed groups (Table 4). Among the non-exposed group, the trend test was not statistically significant. In the exposed group, participants who spent more time outside were more likely to report having a respiratory infection (*p*-value = 0.0004).

## 4. Discussion

This study found a higher prevalence of cough, shortness of breath, hoarseness, respiratory infections, and allergies reported per person among participants living near a coal-burning power plant with coal ash storage facilities compared to a non-exposed group. Additionally, exposed participants reported more respiratory conditions overall. Furthermore, participants living near coal ash storage who spent more time outside were more likely to report having a respiratory infection.

Fugitive coal ash emissions from coal ash storage facilities can contribute to ambient air pollution, which can adversely affect the respiratory system [26]. Coal ash is an emerging environmental threat that can create increased air pollution in communities surrounding coal ash storage sites. The United States Environmental Protection Agency (EPA) has stated that without fugitive dust controls, there could be exceedances of the National Ambient Air Quality Standards for fine particulate matter in the air at residences near coal ash landfills [17].

Inhalable particular matter, which is characterized by the aerodynamic diameter reported in micrometers (μm), are particles less than 10 μm in size, also known as PM_10_. In recent decades, a growing body of epidemiological research has been published on the association between ambient air PM and adverse health outcomes, including those pertaining to respiratory health [16,27,28,29,30]. Research also points to a dose-response relationship as the PM decreases in size; correlation strength increases as the aerodynamic diameter moves from PM_10_ to PM_2.5_ [29,31,32], where PM_2.5_ is defined as particulate matter < 2.5 μm. While PM_10_ and PM_2.5_ both have the potential to cause damage to tissue, PM_2.5_ is capable of traveling deeper into the lungs by penetrating the alveolar gas-exchange region. Here, the particulate matter can enter the blood stream and travel throughout the body.

Limited research has been conducted assessing the respiratory health of populations exposed to coal ash from coal-burning power plants with on-site coal ash storage facilities. Cho, Cho, Shrivastave and Kapre (1994) [33] found that workers exposed to coal ash were more likely to report respiratory effects and Sears and Zierold (2017) [23] found an elevated, but not statistically significant odds ratio for respiratory conditions in children exposed to coal ash compared to children not exposed to coal ash.

The EPA reports that over six million people in the U.S. are exposed to coal ash, but the actual number may be much higher [34]. Regulations overseeing the disposal, storage, and transport of coal ash are minimal. In 2015, coal ash became considered a “special waste” and regulations were passed by the EPA, which set requirements for disposal and storage of coal ash. These regulations included requiring industry to report fugitive dust emissions. However, in 2018, the regulations were rolled back, allowing industry and the individual states to regulate their coal ash. Other countries where coal is a major source of energy have no standards regulating coal ash.

There are several limitations that need to be taken into consideration when discussing the results of this study. First, while the two source populations were comparable according to the census data, there were some differences between participants in the two study groups, specifically the gender ratios and years smoked. Just over half of the non-exposed population reported never smoking compared to 36.3% of the exposed population. Similarly, 25% of the non-exposed population reported smoking for more than 10 years compared with 36.1% of the exposed population. However, we do note that both groups did have similar current smoking rates: 31.9% among the non-exposed group and 34.9% among the exposed group. Differences between the two groups in the number of years smoked could also partially reflect differences in the average age of the two groups. Furthermore, nearly three-fourths of the non-exposed participants were female. It is likely that the comprehensive health clinic had more female visitors, over-representing women in this population. One study examining physician-child interaction reported that 80% of the time, the caretaker with the child was the mother [35], suggesting that women are more likely to accompany their child to a doctor’s appointment than their male counterpart.

Sampling bias may exist. For this study, we wanted to obtain information from participants who lived adjacently to the power plants and coal ash storage facilities, therefore, we targeted recruitment in this population of four neighborhoods. Convenience sampling was used in the neighborhoods adjacent to the power plants. For the non-exposed population, to ensure that we did not get participants who were exposed to coal ash, we took a convenience sample from a local clinic in a region that was situated 60 miles away from the power plants and in a more rural setting.

Another limitation of this study was that a proxy was used for exposure. Since this study was a small pilot study and precursor to a much larger coal ash study, living near the coal ash plant was used as the measure of exposure. The lack of exposure assessment should be taken into consideration when interpreting the results of this study.

This study assessed self-reported behavior and illness; therefore, recall bias is of concern. Healthcare personnel did not verify the respiratory conditions reported by the participants. However, previous research conducted in adult populations has found that self-report is well validated. Researchers have shown that there was high agreement between self-report and medical report for many symptoms and conditions [36,37,38,39]. Additionally, we expect recall bias to have had little effect on the results, as the questionnaire included personal health history recall and long-term habitual behaviors. Furthermore, because some of these behaviors were health-risk behaviors, such as smoking, the accuracy and truthfulness of the results could be of concern [40]. For example, participants may underreport rates of smoking because they believe it is socially undesirable. Both surveys of the exposed and non-exposed populations were anonymous, purposefully excluding personal identifying information in an effort to alleviate this risk.

A final limitation of this study is the potential for selection bias. Participants who may have had more knowledge about coal ash or more respiratory symptoms may have been more likely to participate in the study. In order to attempt to reduce selection bias, we invited all the residents of the four neighborhoods to participate when we were recruiting participants. Additionally, we had tables set-up in central locations so that participants could answer the questionnaire in open spaces in the neighborhoods, where anyone could come and fill out the questionnaire.

One strength is that this study was community-based. Multiple community members helped design the questionnaire and recruit participants. Many researchers have shown that community-based research is improved by working with community members during data collection. This process allows for improved accuracy of results when community members are involved [41,42,43,44,45]. A final strength of the study was that the questionnaire was administered at multiple locations and times, allowing residents to have more opportunities to complete the questionnaire.

## 5. Conclusions

This study is one of the first non-occupational studies to assess respiratory health in adults exposed to coal ash. Although there are several limitations, the results suggest that living near coal ash storage sites could be associated with increased respiratory irritation and illness among the exposed population. Little is known about the health burden that coal ash has on the surrounding community. The results from this study indicate concern and charge future research to assess the impact of coal ash on ambient air pollution and the respiratory health effects. Future studies could help to further assess this relationship by measuring coal ash directly and adjusting for smoking status, years smoked and other potential confounders.

## Figures and Tables

**Table 1:** Characteristics among the non-exposed and exposed populations.

Characteristics	Non-Exposed (*N* = 170)	Exposed (*N* = 231)	*p*-Value
Median Age	43 (164)	52 (203)	0.0004
Gender			<0.0001
Males	25.9% (44)	46.3% (107)	
Females	74.1% (126)	53.7% (124)	
Current Smokers	31.9% (54)	34.9% (80)	0.53
Years Smoked			0.03
Never	52.6% (80)	36.3% (82)	
Less than 1 year	4.6% (7)	3.5% (8)	
1–5 years	8.6% (13)	11.5% (26)	
6–10 years	9.2% (14)	12.4% (28)	
More than 10 years	25.0% (38)	36.3% (82)	
Time Spent Outside			0.46
Never	2.4% (4)	1.7% (4)	
Rarely	3.5% (6)	7.4% (17)	
Sometimes	21.8% (37)	20.0% (46)	
Frequently	31.8% (54)	34.8% (80)	
As much as possible	40.6% (69)	36.1% (83)	
Perception of Health			<0.0001
Excellent	10.2% (17)	0.9% (2)	
Very Good	27.5% (46)	10.5% (24)	
Good	33.5% (56)	41.9% (96)	
Fair	18.6% (31)	30.6% (70)	
Poor	10.2% (17)	16.2% (37)	

**Table 2:** Prevalence of reported respiratory symptoms.

Respiratory Symptoms	Non-Exposed (*N* = 170)	Exposed (*N* = 231)	*p*-Value
Cough			<0.0001
Never	26.4% (43)	4.0% (9)	
Sometimes	64.4% (105)	59.2% (132)	
Frequently	9.2% (15)	36.8% (82)	
Shortness of Breath			<0.0001
Never	43.0% (71)	17.3% (37)	
Sometimes	47.9% (79)	49.1% (105)	
Frequently	9.1% (15)	33.6% (72)	
Hoarseness			<0.0001
Never	68.5% (113)	30.8% (60)	
Sometimes	30.3% (50)	53.3% (104)	
Frequently	1.2% (2)	15.9% (31)	
Respiratory Infections			<0.0001
Never	55.8% (92)	32.1% (61)	
Sometimes	41.8% (69)	51.1% (97)	
Frequently	2.42% (4)	16.8% (32)	
Asthma	26.5% (45)	24.2% (56)	0.61
Allergies	45.3% (77)	52.8% (122)	0.14
Other Lung Diseases	8.8% (15)	18.2% (42)	0.008
Mean Overall Respiratory Health Score	2.82	3.87	<0.0001

**Table 3:** Logistic regression results for respiratory symptoms and health outcomes.

Respiratory Symptoms	Odds Ratio (Confidence Intervals)	Adjusted Odds Ratio ^a^ (Confidence Intervals)
Cough	5.24 (2.90–9.50)	5.30 (2.60–11)
Shortness of Breath	2.65 (1.73–4.07)	2.59 (1.56–4.31)
Hoarseness	3.19 (2.10–4.85)	4.02 (2.45–6.60)
Respiratory Infections	1.68 (1.13–2.51)	1.82 (1.14–2.89)
Asthma	0.89 (0.57–1.41)	1.05 (0.62–1.78)
Allergies	1.35 (0.91–2.01)	*1.62 (1.02–2.58)*
Other Lung Diseases	2.30 (1.23–4.30)	1.63 (0.75–3.55)

^a^ adjusted for gender, age, and years of smoking.

**Table 4:** Gradient response of respiratory infections by time spent outside.

Time Spent Outside	Respiratory Infections			
	Non-Exposed		Exposed	
	No	Yes	No	Yes
Never	0% (0)	4.1% (3)	0% (0)	2.3% (3)
Rarely	1.1% (1)	5.5% (4)	1.6% (1)	9.4% (12)
Sometimes	25.0% (23)	19.2% (14)	14.8% (9)	20.3% (26)
Frequently	32.6% (30)	32.9% (24)	27.9% (17)	39.1% (50)
As much as possible	41.3% (38)	38.4% (28)	55.7% (34)	28.9% (37)
*p*-value for Trend	0.22		0.0004	

## References

[1] Speight J.G. (2015). Handbook of Coal Analysis.

[2] Saikia J., Narzary B., Roy S., Bordoloi M., Saikia P., Saikia B.K. (2016). Nanominerals, fullerene aggregates, and hazardous elements in coal and coal combustion-generated aerosols: An environmental and toxicological assessment. Chemosphere.

[3] Lauer N.E., Hower J.C., Hsu-Kim H., Taggart R.K., Vengosh A. (2015). Naturally occurring radioactive materials in coals and coal combustion residuals in the United States. Environ. Sci. Technol..

[4] United States Energy Information Administration Electricity in the United States—Electricity Explained, Electricity in the United States. https://www.eia.gov/energyexplained/index.cfm?page=electricity_in_the_united_states.

[5] Hock J.L., Lichtman D. (1983). A comparative-study of in-plume and in-stack collected individual coal fly-ash particles. Atmos. Environ..

[6] Flues M., Moraes V., Mazzilli B.P. (2002). The influence of a coal-fired power plant operation on radionuclide concentrations in soil. J. Environ. Radioact..

[7] Liu G., Niu Z., Van Niekerk D., Xue J., Zheng L. (2008). Polycyclic aromatic hydrocarbons (PAHs) from coal combustion: Emissions, analysis, and toxicology. Rev. Environ. Contam. Toxicol..

[8] Patra K.C., Rautray T.R., Tripathy B.B., Nayak P. (2012). Elemental analysis of coal and coal ash by PIXE technique. Appl. Radiat. Isot..

[9] Bednar A.J., Averett D.E., Seiter J.M., Lafferty B., Jones W.T., Hayes C.A., Chappell M.A., Clarke J.U., Steevens J.A. (2013). Characterization of metals released from coal fly ash during dredging at the Kingston ash recovery project. Chemosphere.

[10] Roper A.R., Stabin M.G., Delapp R.C., Kosson D.S. (2013). Analysis of naturally-occurring radionuclides in coal combustion fly ash, gypsum, and scrubber residue samples. Health Phys..

[11] Zibret G., Van Tonder D., Zibret L. (2013). Metal content in street dust as a reflection of atmospheric dust emissions from coal power plants, metal smelters, and traffic. Environ. Sci. Pollut. Res..

[12] Kok V.C., Winn P.R., Hsieh Y.J., Chien J.W., Yang J.M., Yeh G.P. (2019). A pilot study of potentially hazardous trace elements in the aquatic environment near a coastal coal-fired power plant in Taiwan. Environ. Health Insights.

[13] American Coal Ash Association 2017 Coal Combustion Product (CCP) Production and Use Survey Report. https://www.acaa-usa.org/Portals/9/Files/PDFs/2017-Survey-Results.pdf.

[14] Meawad A.S., Bojinova D.Y., Pelovski Y.G. (2010). An overview of metals recovery from thermal power plant solid wastes. Waste Manag..

[15] Shrivastava S., Sahu P., Singh A., Shrivastava L. (2015). Fly ash disposal and diseases in nearby villages: A survey. Int. J. Curr. Microbiol. Appl. Sci..

[16] Ghio A.J., Silbajoris R., Carson J.L., Samet J.M. (2002). Biologic effects of oil fly ash. Environ. Health Perspect..

[17] United States Environmental Protection Agency (2010). Hazardous and solid waste management system; identification and listing of special wastes; disposal of coal combustion residuals from electric utilities; proposed rule. Fed. Reg..

[18] Bencko V., Symon K., Stalnik L., Batora J., Vanco E., Svandova E. (1980). Rate of malignant tumor mortality among coal burning power plant workers occupationally exposed to arsenic. J. Hyg. Epidemiol. Microbiol. Immunol..

[19] Bencko V., Wagner V., Wagnerova M., Batora J. (1988). Immunological profiles in workers of a power plant burning coal rich in arsenic content. J. Hyg. Epidemiol. Microbiol. Immunol..

[20] Liu H.H., Shih T.S., Chen I.J., Chen H.L. (2008). Lipid peroxidation and oxidative status compared in workers at a bottom ash recovery plant and fly ash treatment plants. J. Occup. Health.

[21] Chen H.L., Chen I.J., Chia T.P. (2010). Occupational exposure and DNA strand breakage of workers in bottom ash recovery and fly ash treatment plants. J. Hazard Mater..

[22] Zierold K.M., Sears C.G. (2015). Community views about the health and exposure of children living near a coal ash storage site. J. Commun. Health.

[23] Sears C.G., Zierold K.M. (2017). Health of children living near coal ash. Glob. Pediatr. Health.

[24] Louisville Gas & Electric Cane Run Generating Station. https://lge-ku.com/our-company/community/neighbor-neighbor/cane-run-generating-station.

[25] Louisville Gas & Electric (2010). Assessment of Dam Safety. Coal Combustion Surface Impoundments (Task 3).

[26] Lockwood A., Welker-Hood K., Rauch M., Gottlieb B. (2009). Coal’s Assult on Human Health: A Report from Physicians for Social Responsibility.

[27] Herndon J.M. (2015). Evidence of coal-fly-ash toxic chemical geoengineering in the troposphere: Consequences for public health. Int. J. Environ. Res. Public Health.

[28] Seaton A., MacNee W., Donaldson K., Godden D. (1995). Particulate air pollution and acute health effects. Lancet.

[29] Costa D.L., Dreher K.L. (1997). Bioavailable transition metals in particulate matter mediate cardiopulmonary injury in healthy and compromised animal models. Environ. Health Perspect..

[30] Kampa M., Castanas E. (2008). Human health effects of air pollution. Environ. Pollut..

[31] Dockery D.W., Pope C.A., Xu X., Spengler J.D., Ware J.H., Fay M.E., Ferris Jr B.G., Speizer F.E. (1993). An association between air pollution and mortality in six US cities. N. Engl. J. Med..

[32] Schwartz J. (1994). PM10, ozone, and hospital admissions for the elderly in Minneapolis-St. Paul, Minnesota. Arch. Environ. Health.

[33] Cho K., Cho Y.J., Shrivastava D.K., Kapre S.S. (1994). Acute lung disease after exposure to fly ash. Chest.

[34] Environmental Protection Agency (2015). Hazardous and solid waste management system; disposal of coal combustion residuals from electric utilities (Codified at 40 CFR Parts 257 and 261). Fed. Reg..

[35] Stivers T. (2012). Physician-child interaction: When children answer physicians’ questions in routine medical encounters. Patient Educ. Couns..

[36] Bush T.L., Miller S.R., Golden A.L., Hale W.E. (1989). Self-report and medical record report agreement of selected medical conditions in the elderly. Am. J. Public Health.

[37] Kriegsman D.M., Penninx B.W., van Eijk J.T., Boeke A.J., Deeg D.G. (1996). Self-reports and general practitioner information on the presence of chronic diseases in community dwelling elderly. A study on the accuracy of patients’ self-reports and on the determinants of inaccuracy. J. Clin. Epidemiol..

[38] Happanen N., Miilunpals S., Pasanen M., Oja P., Vuori I. (1997). Agreement between questionnaire data and medical records of chronic diseases in middle-aged and elderly Finnish men and women. Am. J. Epidemiol..

[39] Skinner K.M., Miller D.R., Lincoln E., Lee A., Kazis L.E. (2005). Concordance between respondent self-reports and medical records for chronic conditions: Experience from the Veterans Health Study. J. Ambul. Care Manag..

[40] Brener N.D., Billy J.O., Grady W.R. (2003). Assessment of factors affecting the validity of self-reported health-risk behavior among adolescents: Evidence from the scientific literature. J. Adolesc. Health.

[41] Schulz A.J., Parker E.A., Israel B.A., Becker A.B., Maciak B.J., Hollis R. (1998). Conducting a participatory community-based survey for a community health intervention on Detroit’s east side. J. Public Health Manag. Pract..

[42] Thompson S. (2000). Participatory epidemiology: Methods of the Living with Diabetes Project. Int. Q. Commun. Health Educ..

[43] Green L.W., Mercer S.L. (2001). Can public health researchers and agencies reconcile the push from funding bodies and the pull from communities?. Am. J. Public Health.

[44] Corburn J. (2002). Environmental justice, local knowledge, and risk: The discourse of a community-based cumulative exposure assessment. Environ. Manag..

[45] Tajik M., Minkler M. (2006). Environmental justice research and action: A case study in political economy and community-academic collaboration. Int. Q. Commun. Health Educ..

